# Trend of Mortality Due to Congenital Anomalies in Children Younger Than 5 Years in Eastern China, 2012-2021: Surveillance Data Analysis

**DOI:** 10.2196/53860

**Published:** 2024-06-03

**Authors:** Wen-Hong Dong, Jun-Xia Guo, Lei Wang, Shuang-Shuang Zheng, Bing-Quan Zhu, Jie Shao

**Affiliations:** 1 Department of Child Health Care Children’s Hospital Zhejiang University School of Medicine Hangzhou China; 2 National Clinical Research Center for Child Health Hangzhou China

**Keywords:** under-five years, congenital anomalies, mortality, death cause, rank

## Abstract

**Background:**

As one of the leading causes of child mortality, deaths due to congenital anomalies (CAs) have been a prominent obstacle to meet Sustainable Development Goal 3.2.

**Objective:**

We conducted this study to understand the death burden and trend of under-5 CA mortality (CAMR) in Zhejiang, one of the provinces with the best medical services and public health foundations in Eastern China.

**Methods:**

We used data retrieved from the under-5 mortality surveillance system in Zhejiang from 2012 to 2021. CAMR by sex, residence, and age group for each year was calculated and standardized according to 2020 National Population Census sex- and residence-specific live birth data in China. Poisson regression models were used to estimate the annual average change rate (AACR) of CAMR and to obtain the rate ratio between subgroups after adjusting for sex, residence, and age group when appropriate.

**Results:**

From 2012 to 2021, a total of 1753 children died from CAs, and the standardized CAMR declined from 121.2 to 62.6 per 100,000 live births with an AACR of –9% (95% CI –10.7% to –7.2%; *P*<.001). The declining trend was also observed in female and male children, urban and rural children, and neonates and older infants, and the AACRs were –9.7%, –8.5%, –8.5%, –9.2%, –12%, and –6.3%, respectively (all *P*<.001). However, no significant reduction was observed in children aged 1-4 years (*P*=.22). Generally, the CAMR rate ratios for male versus female children, rural versus urban children, older infants versus neonates, and older children versus neonates were 1.18 (95% CI 1.08-1.30; *P*<.001), 1.20 (95% CI 1.08-1.32; *P*=.001), 0.66 (95% CI 0.59-0.73; *P*<.001), and 0.20 (95% CI 0.17-0.24; *P*<.001), respectively. Among all broad CA groups, circulatory system malformations, mainly deaths caused by congenital heart diseases, accounted for 49.4% (866/1753) of deaths and ranked first across all years, although it declined yearly with an AACR of –9.8% (*P*<.001). Deaths due to chromosomal abnormalities tended to grow in recent years, although the AACR was not significant (*P*=.90).

**Conclusions:**

CAMR reduced annually, with cardiovascular malformations ranking first across all years in Zhejiang, China. Future research and practices should focus more on the prevention, early detection, long-term management of CAs and comprehensive support for families with children with CAs to improve their survival chances.

## Introduction

As a diverse range of structural, functional, and metabolic malformations present at birth, congenital anomalies (CAs) pose a great burden to the families involved [1]. According to the World Health Organization’s latest estimate [2], more than 240,000 newborns die from CAs in the first 28 days of life every year worldwide, and another 170,000 newborns who survive then die from CAs in the following 5 years. Although the world has witnessed encouraging reductions in CA burdens with regard to both mortality and disability-adjusted life-years [1], mortality due to CAs still constitutes 9.4% of all deaths for children younger than 5 years and ranked fourth among all causes globally [3]. For regions such as southern sub-Saharan Africa, the under-5 mortality (U5MR) due to CAs showed no sign of decline; instead, it had an annual increase of 0.17% from 1990 to 2019 [4]. In China’s case, we have also observed an ongoing decline of both U5MR and CA-related mortality [5]. However, deaths caused by CAs remain one of the leading causes of child mortality [6], as observed in other countries [7].

Even if children with CAs survive from an early death, the long-term physical disability or functional disorder still leaves a great burden to both the family and society. It was estimated that the cost of birth defect–related hospitalizations in the United States was US $22.9 billion per year [8]. Although the total medical costs regarding all birth defects is not available in China, the median expenditure of surgery for congenital heart disease (CHD) in China was reported to be CN ¥67,867 (US $9839) in 2020 [9]. For newborns, the median cost was as high as CN ¥144,380 (US $20,931), which was 5.24 times of the median yearly per capita disposable income of the Chinese population in 2020 [9].

According to data issued by the National Bureau of Statistics of China, Zhejiang, a province located in Eastern China, ranked fourth among all 34 administrative regions regarding regional total gross domestic product (GDP) and eighth in GDP per capita in 2022. It has the best medical resources and public health services among the 28 provinces and autonomous regions in the country, excluding the other 6 administrative regions including Beijing, Shanghai, and Hong Kong due to the incomparable size of the targeted service population [10]. However, with the epidemiological transition of the death-cause pattern from infectious diseases to noncommunicable diseases across the whole society [11], little is known about the survival burden of CAs in children younger than 5 years in Zhejiang. Therefore, we conducted this study to understand the trend and burden of under-5 CA mortality (CAMR) in this more economically advanced province in China.

## Methods

In this descriptive study, data from a long-existing mortality surveillance system in Zhejiang were used.

### Mortality Surveillance

Mortality surveillance for children younger than 5 years in Zhejiang started in the early 1990s and is carried out by health workers from 3 levels (village, county, and city) of child health care institutes, mainly maternal and child health care hospitals and community health care centers. Out of the 90 administrative districts and counties, 30 were randomly chosen, and the vital status of children younger than 5 years is routinely monitored in these districts and counties according to the protocol designed by the National Maternal and Child Surveillance Office, as reported elsewhere [5].

Briefly, all deaths that occur in the first 5 years after birth are recorded in a structured, paper-based death report card (DRC) by a physician in a hospital or a community health care worker, depending on whether the death event involves medical treatments in a hospital. Information on the children’s demographics, obstetric status, hospitalization, and death is recorded in the DRC and submitted to the provincial-level surveillance office seasonally, together with a quarterly report including residence- and sex-specific number of live births and deaths in each village.

### Quality Control

To ensure the accuracy of the children’s survival status and the information in the DRC, cross-checking with the local maternal delivery system, vaccination information system, household registration and cancellation system in the Bureau of Public Security, etc, is performed by village-, county-, and city-level health workers quarterly, semiannually, and semiannually, respectively. Provincial-level staff perform a field quality control investigation every year. To minimize information error, 2 experienced physicians were invited to review all DRCs in the data preparation process independently, and any disagreement were settled by a third senior physician.

### CA Groups

Deaths due to CAs (ie, CAs as the underlying cause of death) were coded according to the *International Classification of Diseases, 10th Revision* (*ICD-10*) and recorded in the DRC. In this study, we combined them into 10 broad groups, which were nervous system malformations (Q00-Q07); congenital malformations of the eye, ear, face, and neck (Q10-Q18); circulatory system anomalies including CHD (Q20-Q28); respiratory system malformations (Q30-Q34); cleft lip and cleft palate (Q35-Q37); other digestive system malformations (Q38-Q45); urogenital malformations (Q50-Q64); musculoskeletal malformations (Q65-Q79); chromosomal abnormalities including Down syndrome (Q90-Q99); and other congenital anomalies (Q80-Q89).

### Statistical Analysis

Three age groups were generated during analysis, that is, neonates (aged 0-27 days), older infants (aged 28-364 days), and older children (aged 1-4 years). In the analysis, year-, age-, sex-, and residence-specific CAMRs were calculated as the number of CA deaths in the corresponding year, age group, sex (male or female), and residential region (urban or rural) divided by the number of live births in that specific group. Urban and rural regions were defined according to the fifth digit of the 12-digit administrative code issued by National Bureau of Statistics, for which 1 represents an urban region and all other digits represent a rural region. All mortality data were then standardized by using sex- and residence-specific live birth data from 2020 National Population Census of China. The average annual change rates (AACRs) of CAMR and the rate ratios (RRs) of CAMR for male versus female children, rural versus urban children, older infants versus neonates, and older children versus neonates were analyzed using the Poisson regression model after adjusting for age, sex, and residence when appropriate. All analyses were conducted in SAS (version 9.2; SAS Institute), and a 2-sided *P*<.05 was considered significant.

### Ethical Considerations

The Medical Ethics Committee of Children’s Hospital, Zhejiang University School of Medicine approved this study (2023-IRB-0248-P-01). Since all mortality data were deidentified from the government-established death surveillance system, informed consent was waived by the Medical Ethics Committee.

## Results

From 2012 to 2021, a total of 1753 deaths caused by CAs were reported and the proportion of CA deaths stabilized around 20% of all deaths (Multimedia Appendix 1). The standardized CAMR in Zhejiang dropped from 121.2 per 100,000 live births in 2012 to 62.6 per 100,000 live births in 2021, representing a 48.3% (58.6/121.2) decline in total with a 9% annual reduction on average (*P*<.001; Table 1). Compared with female children, the CAMR of male children was generally higher in each year of the last decade, and the adjusted RR in total was 1.18 (95% CI 1.08-1.30; *P*<.001). Generally, the AACRs for female and male children were –9.7% (95% CI –12.3% to –7.0%; *P*<.001) and –8.5% (95% CI –11.1% to –6.1%; *P*<.001), respectively.

Table 2 shows that relative to children from urban areas, rural children had a 20% higher CAMR (RR 1.20, 95% CI 1.08-1.32; *P*=.001). Moreover, rural children had a higher AACR than their urban counterparts and the AACRs for urban and rural children were –8.5% (95% CI –11.6% to –5.4%; *P*<.001) and –9.2% (95% CI –11.3% to –7.1%; *P*<.001), respectively.

CAMR declined annually by 12% (95% CI 9.6%-14.5%; *P*<.001) for neonates and by 6.3% (95% CI 3.4%-9.2%; *P*<.001) for older infants, but not in older children (*P*=.22; Table 3). Generally, neonates had the highest standardized CAMR compared to older infants and older children (47.2 versus 31.0 and 9.5 per 100,000 live births; Table 3). However, the CAMR gaps across age groups narrowed annually. For older infants, the RR versus neonates turned insignificant since 2018 (all *P*>.05) and increased from 0.48 (95% CI 0.37-0.63; *P*<.001) in 2012 to 1.03 (95% CI 0.65-1.62; *P*=.91) in 2021. For older children, the RR versus neonates grew from 0.17 (95% CI 0.11-0.25) in 2012 to 0.35 (95% CI 0.19-0.66) in 2021 (*P*<.001 in all years).

**Table 1:** Standardized under-5 mortality caused by congenital anomalies in Zhejiang from 2012 to 2021 by sex.

Year	Total^a^		Sex^b^						RR^c,d^ (95% CI)		*P* value
	Deaths, n	Mortality^e^	Female^f^		Male^g^						
			Deaths, n	Mortality	Deaths, n	Mortality					
Total	1753	87.7	757	79.9	992	94.7	1.18 (1.08-1.30)			<.001	
2012	272	121.2	114	108.5	158	132.7	1.22 (0.96-1.56)			.10	
2013	258	119.3	121	118.3	137	120.1	1.02 (0.79-1.30)			.91	
2014	198	93.8	92	91.6	106	95.8	1.05 (0.79-1.39)			.74	
2015	220	104.8	78	79.1	141	128.0	1.64 (1.25-2.17)			<.001	
2016	184	87.2	86	86.3	97	88.0	1.02 (0.76-1.36)			.91	
2017	180	75.3	83	72.8	96	77.6	1.06 (0.79-1.42)			.71	
2018	145	72.1	58	60.3	87	82.6	1.36 (0.98-1.90)			.07	
2019	124	66.5	54	61.5	69	71.1	1.17 (0.82-1.67)			.39	
2020	84	52.7	37	48.8	47	56.3	1.15 (0.75-1.77)			.52	
2021	88	62.6	34	50.6	54	73.3	1.45 (0.94-2.22)			.09	

^a^The annual average change rate (AACR) of under-five mortality caused by congenital anomalies in total from 2012 to 2021 was –9.0% (95% CI –10.7% to –7.2%; *P*<.001).

^b^Four children with unknown sex between 2012 and 2021 were not included in the analysis.

^c^RR: rate ratio.

^d^RR of under-5 mortality due to congenital anomalies for male versus female children after adjusting for age group and residence.

^e^The unit of standardized mortality was per 100,000 live births.

^f^The AACR of under-five mortality caused by congenital anomalies in female children from 2012 to 2021 was –9.7% (95% CI –12.3% to –7.0%; *P*<.001).

^g^The AACR of under-five mortality caused by congenital anomalies in male children from 2012 to 2021 was –8.5% (95% CI –11.1% to –6.1%; *P*<.001).

**Table 2:** Standardized under-5 mortality caused by congenital anomalies in Zhejiang from 2012 to 2021 by residence.

Year	Residence				RR^a,b^ (95% CI)		*P* value
	Urban^c^		Rural^d^				
	Deaths, n	Mortality^e^	Deaths, n	Mortality			
Total	537	78.3	1216	93.3	1.20 (1.08-1.32)	.001	
2012	81	101.8	191	132.9	1.30 (1.01-1.69)	.045	
2013	75	98.9	183	131.5	1.33 (1.02-1.74)	.04	
2014	73	100.7	125	89.6	0.89 (0.67-1.19)	.43	
2015	72	103.0	148	105.9	1.03 (0.77-1.36)	.86	
2016	48	68.1	136	98.6	1.45 (1.04-2.01)	.03	
2017	54	66.0	126	80.9	1.23 (0.89-1.69)	.21	
2018	37	54.8	108	82.4	1.50 (1.04-2.19)	.03	
2019	36	57.1	88	72.2	1.26 (0.86-1.86)	.24	
2020	30	54.8	54	51.5	0.94 (0.60-1.47)	.79	
2021	31	60.7	57	63.7	1.05 (0.68-1.62)	.83	

^a^RR: rate ratio.

^b^RR of under-5 mortality due to congenital anomalies for rural versus urban children after adjusting for sex and age group.

^c^The annual average change rate (AACR) of under-five mortality caused by congenital anomalies in urban children from 2012 to 2021 was –8.5% (95% CI –11.6% to –5.4%; *P*<.001).

^d^The AACR of under-five mortality caused by congenital anomalies in rural children from 2012 to 2021 was –9.2% (95% CI –11.3% to –7.1%; *P*<.001).

^e^The unit of standardized mortality was per 100,000 live births.

**Table 3:** Standardized under-5 mortality caused by congenital anomalies in Zhejiang from 2012 to 2021 by age group.

Year	Age group										Older infants versus neonates					Older children versus neonates		
	Neonates^a^			Older infants^b^			Older children^c^			RR^d,e^ (95% CI)			*P* value		RR (95% CI)			*P* value
	Deaths, n	Mortality^f^	Deaths, n		Mortality	Deaths, n		Mortality										
Total	944	47.2	618		31.0	191		9.5	0.66 (0.59-0.73)			<.001		0.20 (0.17-0.24)			<.001	
2012	165	73.9	79		35.0	28		12.4	0.48 (0.37-0.63)			<.001		0.17 (0.11-0.25)			<.001	
2013	139	64.4	95		43.8	24		11.1	0.68 (0.53-0.89)			.004		0.17 (0.11-0.27)			<.001	
2014	117	55.6	65		30.7	16		7.4	0.56 (0.41-0.75)			<.001		0.14 (0.08-0.23)			<.001	
2015	118	56.4	78		37.1	24		11.3	0.67 (0.50-0.89)			.006		0.21 (0.13-0.32)			<.001	
2016	99	46.7	67		31.8	18		8.6	0.68 (0.50-0.93)			.02		0.18 (0.11-0.30)			<.001	
2017	102	42.4	63		26.8	15		6.2	0.62 (0.46-0.85)			.003		0.15 (0.09-0.26)			<.001	
2018	69	34.4	54		27.0	22		10.7	0.78 (0.55-1.12)			.18		0.32 (0.20-0.52)			<.001	
2019	54	28.8	50		27.0	20		10.8	0.94 (0.64-1.39)			.77		0.38 (0.23-0.63)			<.001	
2020	44	27.3	29		18.5	11		6.9	0.66 (0.41-1.05)			.08		0.25 (0.13-0.48)			<.001	
2021	37	26.3	38		27.1	13		9.2	1.03 (0.65-1.62)			.91		0.35 (0.19-0.66)			<.001	

^a^The annual average change rate (AACR) of under-five mortality caused by congenital anomalies in neonates from 2012 to 2021 was –12% (95% CI –14.5% to –9.6%; *P*<.001).

^b^The AACR of under-five mortality caused by congenital anomalies in older infants from 2012 to 2021 was –6.3% (95% CI –9.2% to –3.4%; *P*<.001).

^c^The AACR of under-five mortality caused by congenital anomalies in older children from 2012 to 2021 was –3.2% (95% CI –8.3% to 2.0%; *P*=.22).

^d^RR: rate ratio.

^e^RR of under-5 mortality due to congenital anomalies adjusted for sex and residence.

^f^The unit of standardized mortality was per 100,000 live births.

**Figure 1:**
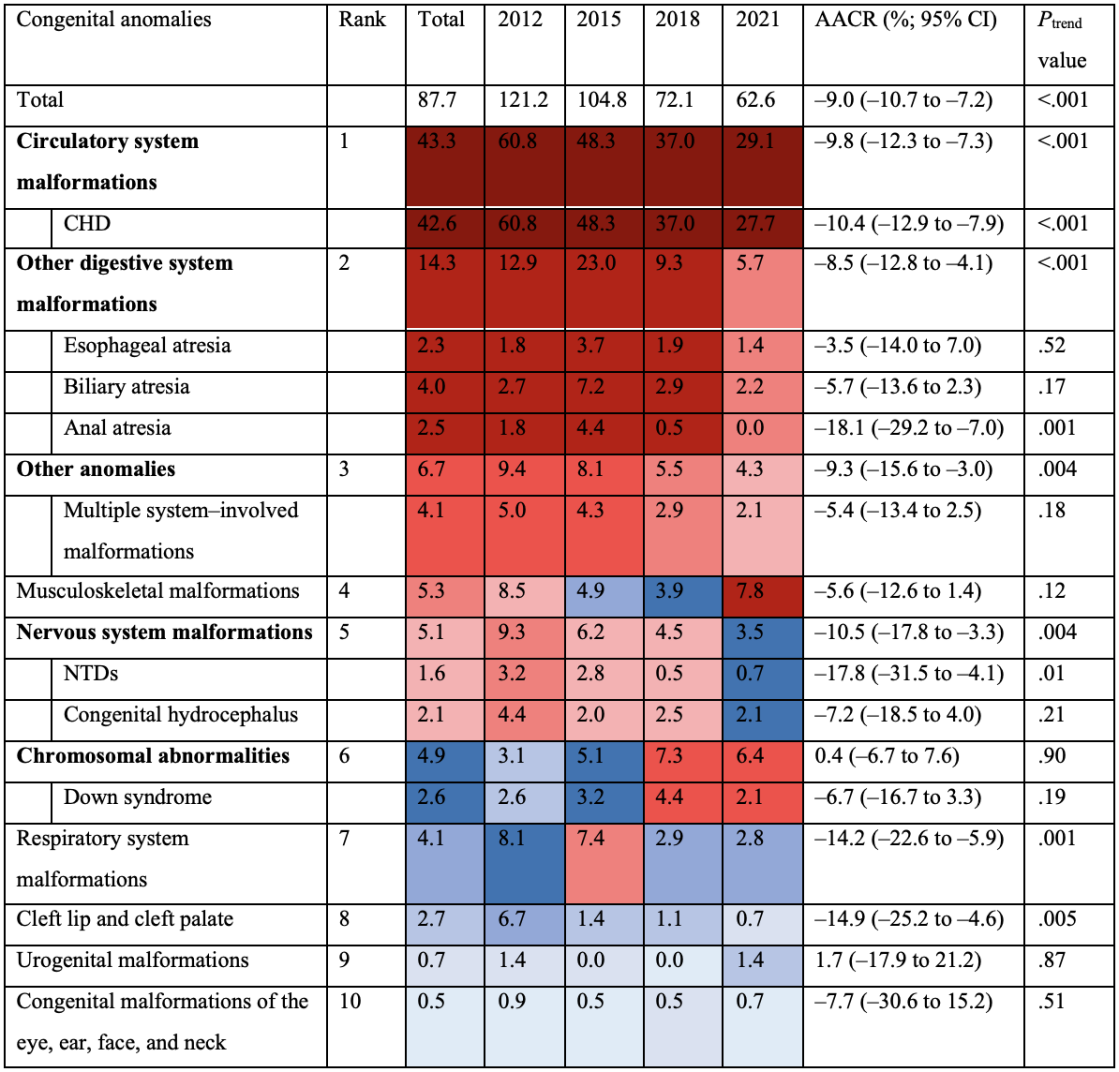
Ranking of standardized under-5 mortality caused by 10 broad congenital anomaly groups and certain common subtypes in Zhejiang from 2012 to 2021. All data in this figure represent standardized under-5 mortalities due to certain congenital anomaly groups or subtypes (unit: per 100,000 live births). Different shades of red and blue indicating the rank of mortality caused by the broad category of congenital malformations: the darker the red is, the higher the rank; and the lighter the blue is, the lower the rank. AACR: annual average change rate; CHD: congenital heart disease; NTD: neural tube defect.

Although a 9.8% (95% CI 7.3%-12.3%; *P*<.001) annual reduction was witnessed in circulatory system anomalies and more than half of the decline (33.1/58.6 per 100,000 live births, 56.5%) in the CAMR was contributed by the reduction in CHD mortality, it still ranked first across all years in the last decade. Apart from CHD, the CAMRs reduced annually for all digestive (*P*<.001), other (*P*=.004), neural (*P*=.004), and respiratory (*P*=.001) anomalies, whereas CAMRs for the other 4 CA groups remained stable (all *P*>.05). Among the 10 CA groups, the rank of causes of death attributed to chromosomal abnormalities changed the most: from the eighth in 2012 to the third in 2021, with a 0.4% (95% CI –6.7% to 7.6%; *P*=.90) insignificant annual increase. Details of the mortality for the 10 CA groups and a few common CA subtypes every 3 years from 2012 to 2021 are depicted in Figure 1.

Multimedia Appendix 2 shows that 944 (53.9%) and 1562 (89.1%) out of 1753 deaths were caused by CAs occurred during the neonatal and infantile period, respectively. Among all common CAs, some were more fatal, with more than 70% of deaths occurring in the first 7 days of life, such as neural tube defects (NTDs; 25/32, 78%) and multiple systems–involved malformations (60/81, 74%). Some were less fatal, with better survival chances over 1 year, such as Down syndrome (17/52, 33%) and biliary atresia (23/81, 28%).

## Discussion

### Principal Findings

In this retrospective study, we observed an annual 9% reduction of CAMR in Zhejiang, and the change was significant in male and female children, urban and rural children, and neonatal and older infants. Generally, CAMRs were higher in male versus female children, rural versus urban children, and neonatal versus older infants. Among all CAs, some are more fatal, with ≥70% of deaths occurring in the first 7 days of life, such as NTDs (78%) and multiple systems–involved malformations (74%), whereas some are less fatal, with more survival chances over 1 year, such as Down syndrome (33%) and biliary atresia (28%). Circulatory system malformations, mainly CHDs, caused the maximum deaths in children younger than 5 years. U5MR caused by chromosomal abnormalities, although insignificant, tended to grow in recent years.

### Comparison With Prior Work

The CAMR we observed in Zhejiang was not only lower than most provinces in China [6] but also lower than that in high-income countries such as the United States [12] and European countries [7]. The rather low CAMR and an ongoing declining trend can be explained by several reasons. First, there has been a series of nationally covered maternal and children health programs with stable financial investment from the Chinese government [13]. Among them, some aimed at reducing the prevalence of certain CAs and adverse birth outcomes, such as providing free folic acid tablets to women of reproductive age [14], whereas others aimed at improving the survival and health of children, such as providing antenatal and postnatal health care and health management for children aged 0-6 years [13].

Second, the rapid development and universal application of prenatal and newborn screening is another potential reason for the continuous decline of CAMR in Zhejiang. In 2000, only 7.6% children with Down syndrome were identified prenatally in China [15]. From 2013 to 2017 in Zhejiang, 91%, 96.9% and 100% of Down syndrome, encephalocele, and anencephaly were identified through prenatal screening, respectively [16]. Along with the extensive application of prenatal screening was a ≥90% rate of early termination of pregnancy, leading to less live births with severe CAs [17]. Newborn screening, a well-established public health measure to reduce early infant deaths, is widely used in Zhejiang, and more than 400,000 newborns are screened every year [18].

Third, with the development of postpartum surgical and repair techniques, the survival chances of children with CAs such as CHD, cleft lip, and cleft palate improved greatly [19,20]. For example, although the birth prevalence of CHD increased continuously worldwide [21] as well as in China [22], mortality due to CHD decreased gradually [23]. According to a hospital-based study, in-hospital mortality for children who underwent CHD surgeries during the first month after delivery reduced from 16.4% in the period from 2004 to 2008 to 5% in the period from 2014 to 2018 [24].

Similar to previous studies [6,25], we identified a larger CA death burden in male children than in female children. Both biological and genetic factors may play a role. First, male children are more likely to be born prematurely, which signifies lower survival chances [26]. Second, the X chromosome carries more genes responsible for immune function, leaving male children with CAs more vulnerable to infections [27]. The mortality differences across age groups are mainly related to the type and severity of CAs. As mentioned above, most severe CAs such as NTDs would experience pregnancy termination, and less than half of live births with NTDs would survive over 1 year [28]. For CAs that are less fatal such as Down syndrome, the life expectancy has increased dramatically and reached 60 years in 2002 [29].

The high death burden caused by CHD in our study was prevalent globally [4]. Although great progress has been made, the elimination of CHD remains a great challenge. On the one hand, as a public health issue driven mainly by genetics, it is difficult to remove the genetic predisposition and other environmental contributors that once existed [30]. On the other hand, timely diagnosis and treatment is still an issue to be solved. In Western countries such as Norway, only 13% of infants were discharged without the identification of severe CHDs [31], whereas in China, this number was 52.5% and can be as high as 71% for neonates with asymptomatic critical CHD [32]. In addition, mortality from complex CHD is still higher than other CHDs [33]. Besides, the high medical cost and parental worries for children’s future quality of life might also affect parents’ decision-making on whether to take further treatment or not.

In our study, we also noticed an increase in U5MR for chromosomal issues. Although insignificant, several reasons may provide an explanation. First, the proportion of pregnancies with advanced maternal age, a well-established risk factor of chromosomal abnormalities in the offspring [34], almost doubled after the termination of the One-Child Policy [35]. Second, new genetic techniques promote the detection and correct categorization of chromosomal issues [36]. Third, although detectable, systematic treatment strategies for most abnormalities are still absent [37]. Considering the factors mentioned above, higher prevalence and mortality of chromosomal abnormalities are projected in the future, and more attention should be paid to this issue.

### Limitations

There are mainly 3 limitations in our study. First, the miscoding of death cause might exist. However, we have invited 2 experienced physicians to independently review all DRCs in the data preparation process to minimize this type of error in addition to routine quality control measures. Second, detailed information regarding each live birth with CAs and their survival status in the next 5 years was not collected in our work, limiting further analysis and comparisons on survival chances for different CA subtypes and the associated factors. Third, the analysis for CAs as a contributory cause of study was necessary but not feasible due to the lack of information in this study. It was reported that only 70% of deaths in infants with CAs recorded a CA as the underlying cause of death [38], meaning that the actual burden of CAs is greater than what we found.

### Conclusion

In our study, we observed an ongoing decline in the CAMR in Eastern China, with cardiovascular malformations ranking first across all years. Despite the remarkable achievements, we still face great challenges, and we hereby make the following suggestions: (1) researchers should perform more well-designed studies to understand CAs’ risk factors and take measure accordingly to lower CA incidence, similar to what have been achieved with NTDs; (2) we must strengthen in-service education and training of medical staffs to improve their skills and promote “early detection, early diagnosis and early treatment”; (3) more sensitive and population-wide screening techniques such as echocardiography for early identification of CHD should be developed and applied to the public for free to lower undiagnosed CA cases [39]; and (4) we should provide easily accessible educational, emotional, and financial support to families with children with CAs, as reported by Wray and colleagues [40], to increase families’ capabilities and confidence in caring for children with CAs and improve their survival possibilities.

## Appendix

Multimedia Appendix 1 Standardized under-5 mortality and percentages caused by congenital anomalies in Zhejiang from 2012 to 2021.

Multimedia Appendix 2 Age distribution of deaths due to 10 broad congenital anomaly groups and certain common subtypes for children younger than 5 years in Zhejiang from 2012 to 2021.

## Abbreviations

AACR: average annual change rate
CA: congenital anomaly
CAMR: under-5 congenital anomaly mortality
CHD: congenital heart disease
DRC: death report card
GDP: gross domestic product
*ICD-10*: *International Classification of Diseases, 10th Revision*
NTD: neural tube defect
RR: rate ratio
U5MR: under-5 mortality

## Data Availability

The data sets generated and/or analyzed during this study are not publicly available due to safety and privacy concerns but are available from the corresponding author on reasonable request.
