# Supplementary figures and images for: Trend of Mortality Due to Congenital Anomalies in Children Younger Than 5 Years in Eastern China, 2012-2021: Surveillance Data Analysis
Source: JMIR Public Health Surveill. 2024 Jun 3;10:e53860. doi: 10.2196/53860 (PMC11184267; doi:10.2196/53860)

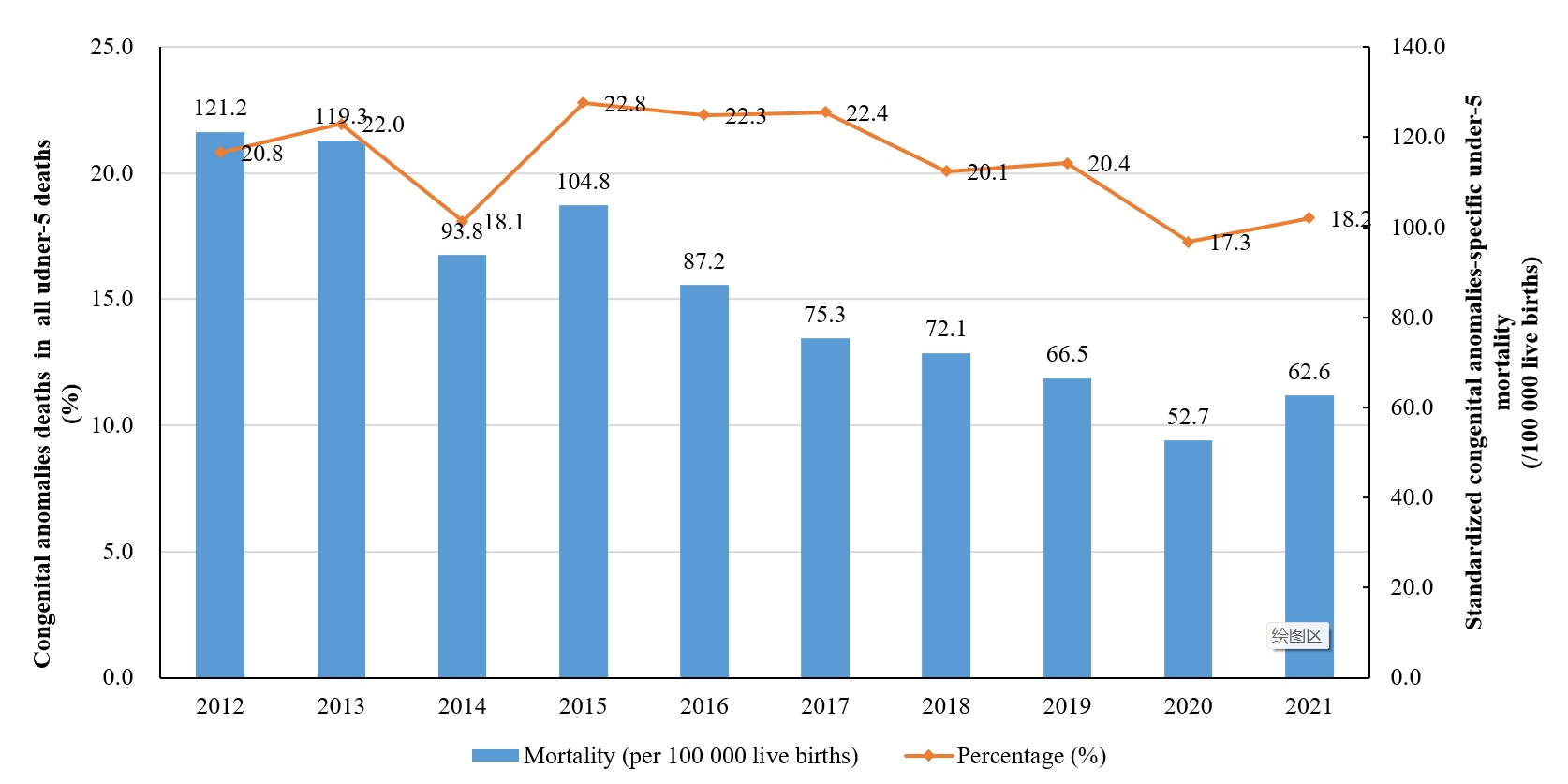

Supplement: Multimedia Appendix 1 [file publichealth_v10i1e53860_app1.png]

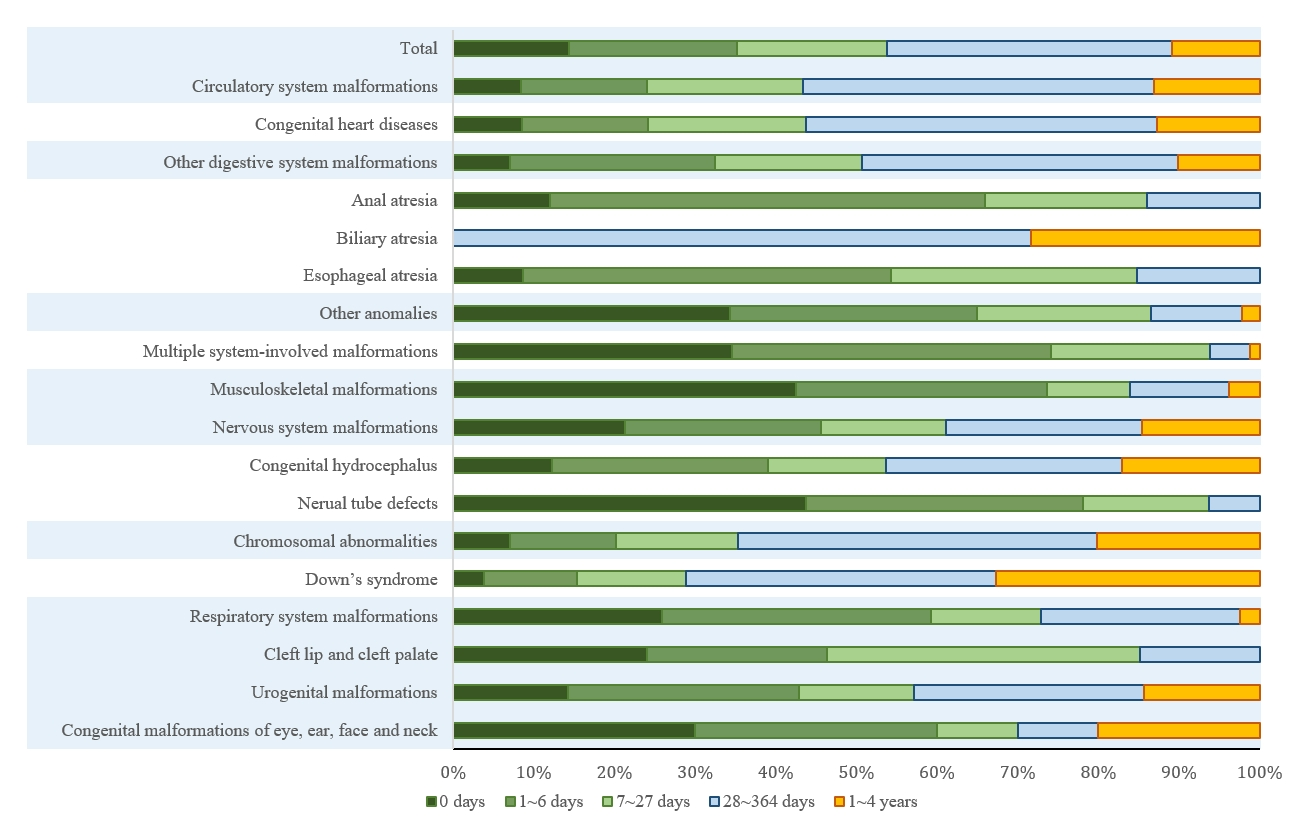

Supplement: Multimedia Appendix 2 [file publichealth_v10i1e53860_app2.png]
